# Egyptian Pediatric Guidelines for the Management of Children with Isolated Thrombocytopenia Using the Adapted ADAPTE Methodology—A Limited-Resource Country Perspective

**DOI:** 10.3390/children11040452

**Published:** 2024-04-09

**Authors:** Galila Mokhtar, Ashraf Abdelbaky, Amira Adly, Dina Ezzat, Gehan Abdel Hakeem, Hoda Hassab, Ilham Youssry, Iman Ragab, Laila M. Sherief, Marwa Zakaria, Mervat Hesham, Niveen Salama, Nouran Salah, Rasha A. A. Afifi, Rasha El-Ashry, Sara Makkeyah, Sonia Adolf, Yasser S. Amer, Tarek E. I. Omar, James Bussel, Eman Abd El Raouf, Mervat Atfy, Mohamed Ellaboudy, Ivan Florez

**Affiliations:** 1 Pediatric Hematology and Oncology Unit, Pediatric Department, Ain Shams University, Cairo 11566, Egypt; galmokhter@med.asu.edu.eg (G.M.); amiraadly73@med.asu.edu.eg (A.A.); hragab68@med.asu.edu.eg (I.R.); smakkeyah@med.asu.edu.eg (S.M.); drmohamed_allaboudy@med.asu.edu.eg (M.E.); 2 Pediatric Department, Faculty of Medicine, Ain Shams University, Cairo 11566, Egypt; shrafabdelbaky@med.asu.edu.eg; 3 Pediatric Hematology Unit, Pediatric Department, Beni-Suef University, Beni-Suef 62521, Egypt; dina.ezzat.ams@o6u.edu.eg; 4 Pediatric Department, October 6 University, Giza 12585, Egypt; 5 Pediatric Hematology and Oncology Unit, Pediatric Department, Minia University, Minia 61519, Egypt; gehan.khalifa@mu.edu.eg; 6 Pediatric Hematology and Oncology Unit, Pediatric Department, Faculty of Medicine, Alexandria University, Alexandria 21526, Egypt; hoda.hassab@alexmed.edu.eg; 7 Pediatric Hematology and Bone Marrow Transplantation Unit, Pediatric Department, Cairo University, Giza 12613, Egypt; ilhamyoussry@kasralainy.edu.eg (I.Y.); niveensab@cu.edu.eg (N.S.); rasha.abdelaziz@kasralainy.edu.eg (R.A.A.A.); eraouf@kasralainy.edu.eg (E.A.E.R.); 8 Pediatric Hematology and Oncology Unit, Pediatric Department, Zagazig University, Zagazig 44519, Egypt; lamesh@zu.edu.eg (L.M.S.); mzmustafa@zu.edu.eg (M.Z.); mahesham@medicine.zu.edu.eg (M.H.); mervatatfy@medicine.zu.edu.eg (M.A.); 9 Pediatric Hematology and Oncology Unit, Pediatric Department, Mansoura University, Mansoura 35516, Egypt; rashaashry@hotmail.com; 10 Pediatric, Hematology Department, Institute of Medical Research and Clinical Studies, National Research Center, Giza 1770, Egypt; sa.habib@nrc.sci.eg; 11 Pediatrics Department, Quality Management Department, King Saud University Medical City, Riyadh 11451, Saudi Arabia; yamer@ksu.edu.sa; 12 Research Chair for Evidence Based Health Care and Knowledge Translation, King Saud University, Riyadh 11451, Saudi Arabia; 13 Department of Internal Medicine, Ribeirao Preto Medical School, University of Sao Paulo (FMRP-USP), Ribeirao Preto 14040-900, SP, Brazil; 14 Pediatrics Department, Faculty of Medicine, Alexandria University, Alexandria 21526, Egypt; tarek.omar@alexmed.edu.eg; 15 Pediatrics Department, Well Cornell Medical College, New York, NY 10065, USA; jbussel@med.cornell.edu; 16 Department of Pediatrics, University of Antioquia, Medellin 050010, Colombia; ivan.florez@udea.edu.co

**Keywords:** bleeding, isolated thrombocytopenia, pediatric

## Abstract

Background: Thrombocytopenia is a prevalent presentation in childhood with a broad spectrum of etiologies, associated findings, and clinical outcomes. Establishing the cause of thrombocytopenia and its proper management have obvious clinical repercussions but may be challenging. This article provides an adaptation of the high-quality Clinical Practice Guidelines (CPGs) of pediatric thrombocytopenia management to suit Egypt’s health care context. Methods: The Adapted ADAPTE methodology was used to identify the high-quality CPGs published between 2010 and 2020. An expert panel screened, assessed and reviewed the CPGs and formulated the adapted consensus recommendations based on the best available evidence. Discussion: The final CPG document provides consensus recommendations and implementation tools on the management of isolated thrombocytopenia in children and adolescents in Egypt. There is a scarcity of evidence to support recommendations for various management protocols. In general, complete clinical assessment, full blood count, and expert analysis of the peripheral blood smear are indicated at initial diagnosis to confirm a bleeding disorder, exclude secondary causes of thrombocytopenia and choose the type of work up required. The International Society of Hemostasis and thrombosis–Bleeding assessment tool (ISTH-SCC BAT) could be used for initial screening of bleeding manifestations. The diagnosis of immune thrombocytopenic purpura (ITP) is based principally on the exclusion of other causes of isolated thrombocytopenia. Future research should report the outcome of this adapted guideline and include cost-analysis evaluations.

## 1. Introduction

**Adapted from Source Guidelines:**

- American Society of Hematology 2019 guidelines for immune thrombocytopenia [1].
- The European guideline on management of major bleeding and coagulopathy following trauma: fifth edition [2].
- Management of severe perioperative bleeding: guidelines from the European Society of Anesthesiology [3].
- Fetal and neonatal alloimmune thrombocytopenia: recommendations for evidence-based practice, an international approach, 2019 [4].
- Guidelines on transfusion for fetuses, neonates and older children [5].
- Guidelines for the Laboratory Investigations of heritable disorders of platelet function [6].
- Updated international consensus report on the investigation and management of primary immune thrombocytopenia [7].

Thrombocytopenia, defined as a platelet count of less than 100 × 10^9^/L, can be acquired or inherited [8,9]. Isolated thrombocytopenia is defined as a low platelet count in the absence of abnormalities of the leukocytic and erythrocytic lineages in the absence of signs or symptoms of systemic illness. The most prevalent etiologies are immune thrombocytopenic purpura (ITP), drug-induced ITP, and inherited thrombocytopenias [10,11].

Neonatal thrombocytopenia is a prevalent morbidity, involving as many as 22–35% of all admissions in neonatal intensive care units [12,13], particularly in very low birth weight, preterm neonates in which a prevalence of thrombocytopenia, 70–80%, has been reported [14,15]. In preterm neonates, early-onset thrombocytopenia (<72 h) is usually secondary to antenatal causes; and often resolves without complications or need for treatment. By contrast, late-onset thrombocytopenia in preterm neonates (>72 h) is nearly always due to post-natal acquired bacterial infection and/or necrotizing enterocolitis, which rapidly leads to severe thrombocytopenia (platelet count < 50 × 10^9^/L). Hypogammaglobulinemia (by facilitating infections) and liver disease are also causes of late-onset thrombocytopenia. Thrombocytopenia is much less common in term neonates and the most important cause is neonatal allo-immune thrombocytopenia (NAIT) [16]. It should be suspected particularly if the day 1 count is 50,000/μL. A variety of new preventive and therapeutic approaches are evolving for fetal and neonatal allo-immune thrombocytopenia (FNAIT) [17].

Establishing the cause of thrombocytopenia in children is sometimes challenging. An individual with newly diagnosed thrombocytopenia is more likely to have an acquired disorder rather than an inherited genetic mutation but this may be difficult to ascertain if no previous blood counts exist. The diverse pathophysiologic causes of acquired thrombocytopenia include increased platelet consumption, autoimmune diseases, splenomegaly with increased intrasplenic platelet pooling, and bone marrow suppression [18].

Two important clinical characteristics of inherited thrombocytopenias are the younger age of presentation and the often stable chronicity/duration of symptoms. Moreover, the presence of a positive family history and symptoms or signs of different conditions, e.g., TAR (absent radii) and WAS (male, eczema, infections), might be a clue. In the event of severe thrombocytopenia, the condition is usually recognized during the perinatal period. Milder disorders are noted sporadically at times of hemostatic stress (e.g., onset of menses) [19]. Immune thrombocytopenia (ITP) is relatively common in childhood, with an annual incidence of 1.9–6.4 per 100,000 children [20]. Khalifa et al. reported a 30% rate of chronic ITP in a large Egyptian study, similar to international reports [21]. There was no gender preference in most newly diagnosed ITP studies; however, chronic ITP was more frequent in females in Egypt [22]. A retrospective report from Lebanon indicated a much lower chronicity rate of 10% [23], while in the TIKI trial from the Netherlands, it ranged from 10% to 12% [24]. Diagnosis of primary ITP is made when isolated thrombocytopenia occurs in the absence of identifiable and specific precipitants [25]. Differential diagnosis includes infectious, immunologic causes, hematologic, endocrine, and neoplastic causes [26,27,28,29,30].

ITP has been described following several viral infections, including hepatitis B, hepatitis C, cytomegalovirus, varicella zoster virus, human immunodeficiency virus, zika virus and COVID-19 virus [31]. Several cases of ITP secondary to COVID-19 have been reported [32,33,34]. Suggested pathomechanisms include the cross-reactivity of the virus-induced antibodies with normal platelets leading to platelet destruction [31].

Several hundred therapeutic agents have been implicated in drug-induced thrombocytopenia. Suggested pathomechanisms include bone marrow toxicity or immune-mediated platelet destruction [35].

Clinical Practice Guidelines (CPGs) have generally recommended a minimum evaluative process to look for secondary causes of thrombocytopenia before the diagnosis of ITP [7]. Medical history should include the type and severity of bleeding, systemic symptoms, history of respiratory infections, recent live viral vaccine, medications, presence of bone pain, and family history of bleeding disorders as well as family history of thrombosis (in case of treatment). Clinical examination should include observation for any dysmorphic features, especially skeletal anomalies, and the presence or absence of hepatosplenomegaly and/or lymphadenopathy [36]. Platelet size could be a clue since ITP often has relatively large platelets, but not as large as the clear majority of inherited thrombocytopenias. Moderate (instead of severe) thrombocytopenia at initial presentation, non-response to first-line treatments, failure to reach resolution of the ITP, and development of new symptoms or laboratory abnormalities would raise suspicion of other causes [37]. There is no single hematologic or biochemical test that is conclusive of a given cause of thrombocytopenia [38]. Reaching the proper diagnosis of thrombocytopenia requires thoughtful and profound clinical and laboratory assessments. In all, even in typical cases, periodic complete blood count (CBC) and review of the blood smear are recommended to exclude the evolution of serious bone marrow or other hematologic disorders until the diagnosis is clear or recovery has occurred [7].

In children, the symptoms of ITP are considered to have a greater impact on the treatment decision than the platelet count. The expectant watch-and-wait for policy is the most common policy in most children with ITP, provided there are caring families with close monitoring [7]. Studies suggest a risk from 0% to 4% of children with newly diagnosed ITP to have severe bleeding requiring intervention [35], with incidence of intracranial hemorrhage from 0% to 1% [39]. Multiple factors should be taken into consideration when deciding to treat or not including bleeding symptoms, especially hematuria, platelets count, existence of headache, recent trauma, medication use, and psychosocial and lifestyle factors [7]. The goal of therapy is to reach a safe platelet count in the absence of bleeding, not a normal platelet count [10].

Despite the presence of numerous publications on the diagnosis and treatment of isolated thrombocytopenia, only a few updated eligible high-quality CPGs provide evidence-based recommendations on clinical issues assessed by expert working groups in the management of isolated thrombocytopenia in children. Unfortunately, these high-quality evidence-based CPGs cannot be applied to the clinical practice in Egypt directly, considering differences in the health system, culture, social background, resources, availability and expenses of treatments, ethnicity, and preference of patients, parents, or guardians. Therefore, currently, available international CPGs warrant appropriate adaptation to facilitate the management of isolated thrombocytopenia in children in Egypt, integrating evidence from Egypt, considering opinions of clinical experts, and following a CPG formal adaptation framework: The Adapted ADAPTE [24,40,41].

Hence, this adapted CPG was prepared, aiming to provide a differential diagnosis and approach to the etiology of bleeding (with focus on thrombocytopenia) in neonates, infants, children, and adolescents in Egypt. The most important goal was to identify neonates, infants, children, and adolescents at high risk of bleeding, providing them with timely cost effective proper management and preventing further bleeding episodes.

## 2. Materials and Methods

This CPG adaptation project was part of the fourth wave of the EPG national CPG projects [42,43]. The Egyptian Pediatric Clinical Practice Guidelines Committee (EPG) Hematology Group, that included 17 members with recognized clinical and research expertise in pediatric hematology representing 9 tertiary research centers from different Egyptian governorates (Ain Shams University, Alexandria University, Armed Forces College of Medicine, Beni-Suef University, Cairo University, Mansoura University, Minia University, National Research Center and Zagazig University) selected this topic as one of the high-priority health topics in Pediatric Hematology in Egypt.

**Figure 1:**
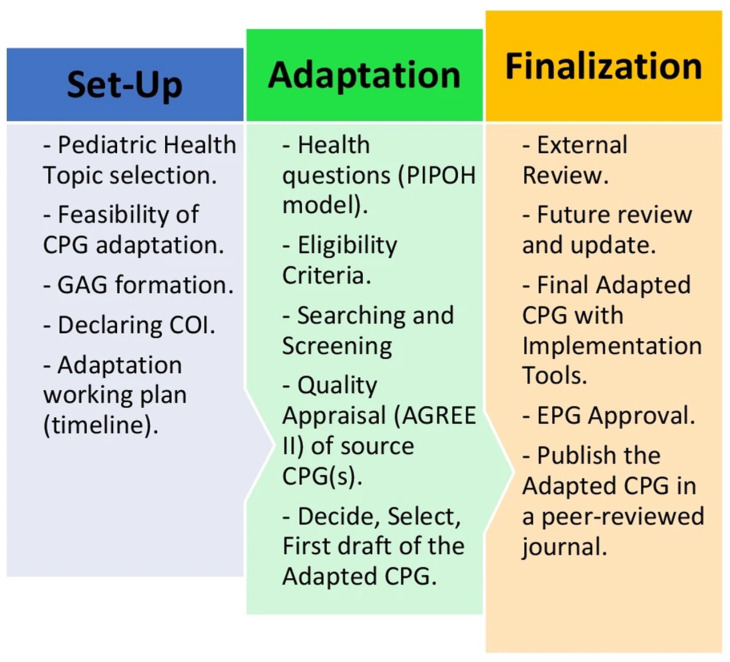
Graphical representation of the Adapted ADAPTE guideline adaptation process. The ‘Adapted ADAPTE’ is divided into three phases: (1) the setup phase, (2) the adaptation phase, and (3) the finalization phase. Abbreviations: AGREE II—The Appraisal of Guidelines for Research and Evaluation II Instrument, CPGs—clinical practice guidelines, COI—conflict of Interests, EPG—Egyptian Pediatric Clinical Practice Guidelines Committee, and GAG—Guideline Adaptation Group [44].

The Adapted ADAPTE CPG formal adaptation method was used for the adaptation process, which included three phases (setup, adaptation, and finalization), nine modules, and 24 steps with modifications in the steps and tools to suit the local general health care setting in Egypt (Figure 1) [24].

A literature search of the relevant CPG and bibliographic electronic database PubMed was performed in July 2022 for relevant source original CPG retrieval. The following search terms were used: “childhood thrombocytopenia”, “pediatric thrombocytopenia”, “immune thrombocytopenic purpura”, “idiopathic thrombocytopenic purpura”, “autoimmune thrombocytopenic purpura” and “isolated thrombocytopenia”, “neonatal thrombocytopenia” and “inherited thrombocytopenia”. Corresponding MeSH terms were used to search for titles and abstracts. Inclusion and exclusion criteria were identified. The search was restricted to articles published from July 2010 to July 2022 to capture evidence-based consensus CPGs in that 10-year period. The following filters were applied: humans, English, evidence-based guidelines, and practice guidelines. Research articles and reviews of the literature were not included. The search results revealed 12 eligible source CPGs that were assessed for methodological quality by six independent reviewers using the Appraisal of Guidelines for Research and Evaluation (AGREE) II Instrument [44,45,46,47].

This was followed by registration of the adapted guideline on the PREPARE (Practice Guideline Registry for transparency) Platform with registration number (PREPARE-2022CN791) [48,49]. Three experts in CPG methodology participated in the Guideline Adaptation Group (GAG).

Seven CPGs met an AGREE II Overall Assessment score of more than 70%, with a score of more than 70% in domain 2 alone, and hence were included as source CPGs for the adaptation [1,2,3,4,5,6,7], while 5 CPGs did not meet the score and were not included (Table 1) [50,51,52,53,54]. Official approvals for adaption from the selected CPG authors were obtained.

Identification and phrasing of the health questions that needed to be tackled were performed using the PIPOH model (Table 2). This was followed by formulation of the adapted recommendations according to the identified health questions. Twenty eight health questions were prioritized (twelve for diagnosis, ten for treatment and six for prevention), for which appropriate recommendations from the adapted CPGs were sought. Evidence levels and grades of recommendation adopted by the EPG were used to classify the quality of evidence and strength of recommendations (Table 3 and Table 4) [7]. Twelve implementation tools supporting the recommendations were adopted and added to the CPG book. The external review process included consultation of 3 national and 1 international clinical reviewers, and 1 international guideline methodology reviewer. Their feedback was incorporated in the finalized adapted CPG with the set of implementation strategies and 12 implementation tools including the bleeding assessment score, an etiology table, a laboratory thresholds table, two clinical algorithms, two bleeding assessment tools, a differential diagnosis tool, a diagnostic tool, a medication table, a platelet transfusion threshold guide, and health education guidance for patients and families in Arabic. Given the rate of development of new treatments for idiopathic thrombocytopenia, the EPG hematology group will review the need for updates every 5 years and will be guided by the Checklist for the Reporting of Updated Guidelines (CheckUp) Tool [55].

The components of the adapted CPG were further reported using the Reporting Items for Practice Guidelines in Healthcare (RIGHT) extension statement for the reporting of adapted CPGs (RIGHT-Ad@pt Checklist) [56,57] (Supplementary Materials). Adaptation approval was taken from the adapted guidelines authors.

## 3. Results

Recommendations:

1. Diagnosis of thrombocytopenia
  1.1. What is a validated bleeding score used for neonates?

The NeoBAT is a modified WHO bleeding assessment tool to record neonatal bleeding episodes to standardize the clinical recording of bleeding in premature and term neonates in an intensive care setting [58] (C).

  1.2. What is the initial evaluation for neonates presenting with bleeding and thrombocytopenia?

An evaluation of patients with abnormal bleeding requires objective clinical assessment of bleeding history, any family history, history of maternal drug intake, history of perinatal sepsis and physical examination for any physical anomalies or organomegaly, and any associated morbidity (C).

Laboratory investigations of platelet number are recommended in any patient where bleeding symptoms are not fully explained by standard clinical laboratory investigations (C).

  1.3. What is the initial evaluation for fetal/neonatal alloimmune thrombocytopenia (FNAIT)?

A cranial ultrasound should be performed to screen for intracranial hemorrhage (ICH) in all neonates suspected of fetal/neonatal alloimmune thrombocytopenia (FNAIT) within 24 h of delivery (1B).

FNAIT testing should include (1B):

HPA genotyping from the mother and the father.

Alloantibody testing of maternal serum.

A crossmatch with paternal platelets.

A neonate with FNAIT should have platelet counts monitored until the platelets are normal in the absence of treatment (1C).

  1.4. What are the antenatal diagnostic tests for possible FNAIT?

Fetal HPA typing, preferably using non-invasive methods, if adequate quality is assured, should be performed during pregnancy when the father is unknown, unavailable for testing or heterozygous for the implicated antigen. Only HPA-1ab can currently be typed using cell-free fetal DNA.

Balance of harms and benefits: The alternative is amniocentesis, which is associated with a very small risk of fetal demise (1B).

  1.5. What are the points in history and examination suggestive of inherited thrombocytopenia in children?

Thrombocytopenia has been present since early life; no normal count is known.

Platelet counts (if any available) have been relatively stable.

A positive family history for a similar disorder.

Characteristic physical features are present.

Failure to respond to first-line ITP treatment.

Cytoplasmic inclusions in the neutrophils of some pathological forms

Platelets are very large (C).

  1.6. What are the tests required to exclude inherited thrombocytopenia?

Mean platelet volume may be used to differentiate ITP from inherited thrombocytopenia; increased mean platelet volume can be suspected on smear if there are large platelets (2B). This may be subjective unless the platelets are very large, strongly suggesting an inherited thrombocytopenia.

Platelet function testing is not correlated with clinical bleeding unless it secures a specific diagnosis, e.g., Glanzmann, Bernard–Soulier (C).

Flow cytometry should be used in the investigation or confirmation of Bernard–Soulier syndrome by a moderate to marked reduction in CD42a (GpIX) and CD42b (Gp1bα) (1C).

Flowcytometry may also be used to investigate abnormalities in the collagen (GpVI and GpIa/IIa), thrombin receptors (PAR-1) and Glanzmann thrombasthenia (GpIb, GpIIb and GpIIIa) (1B).

VWF multimer analysis or the VWF collagen binding/VWF antigen ratio should be used if VWD type 2B is suspected (C).

  1.7. What is the initial evaluation for children and adolescents presenting with bleeding and thrombocytopenia?

A complete history, physical examination, family history, full blood count, and expert analysis of the peripheral blood smear should be performed and carefully evaluated at initial diagnosis to confirm a bleeding disorder, exclude secondary causes of thrombocytopenia and choose the type of work up required. (C).

  1.8. What is a validated general bleeding score used for children?

The International Society of Hemostasis and thrombosis–Bleeding assessment tool (ISTH-SCC BAT) comprises 14 categories for assessing bleeding symptoms could be used for initial screening of bleeding manifestations (1C).

  1.9. What are the diagnostic criteria for immune thrombocytopenia?

The diagnosis of ITP is based principally on the exclusion of other causes of isolated thrombocytopenia using patient history, physical examination, blood count, and evaluation of the peripheral blood film (to exclude other hematological conditions) (2B).

In isolated thrombocytopenia with no abnormal physical findings and no abnormalities in the blood count or on blood smear, a bone marrow examination is not required in the initial diagnosis, whether or not treatment is recommended (B).

  1.10. What are the additional diagnostic tests required in children and adolescents with ITP?

Quantitative immunoglobulin (Ig) level testing is indicated to exclude an immune deficiency syndrome even in the absence of past history of infections or before treatment with IVIg; ideally the test should be sent but the results do not need to be available for IVIg to be initiated (C).

Testing for antinuclear antibodies is not necessary in the evaluation of children and adolescents with newly diagnosed ITP but is most appropriate in adolescent females with ITP (1B).

  1.11. What are the indications of bone marrow examination in patients with ITP?

Newly diagnosed ITP in children:

Bone marrow examination is unnecessary in children and adolescents with the typical features of ITP (1B).

Bone marrow examination is necessary in children who fail to respond to IVIG therapy (1B).

Bone marrow examination is not necessary in patients prior to initiation of treatment of corticosteroids if all criteria for ITP are met (2C).

Bone marrow examination could be appropriate in those relapsing after remission, in patients not responding to initial treatment options, where splenectomy is considered, or if other abnormalities are detected in the blood count (bicytopenia or pancytopenia) or morphologic abnormalities (3C).

A bone marrow examination should ideally include all studies including aspirate, biopsy, flow cytometry, and cytogenetics (C).

  1.12. What are the subsequent investigations in children and adolescents with persistent or chronic ITP?

A direct antibody test is recommended to exclude coexistent autoimmune hemolytic anemia, especially prior to therapy; a reticulocyte count may also be indicated.

Immunoglobulin levels.

Lupus and other markers of autoimmune diseases that might require specific treatment (e.g., test for antiphospholipids, antibodies, antinuclear antibodies, anticardiolipin antibody, lupus anticoagulant) and thyroid testing.

Chronic infections ideally by PCR (hepatitis A, B, and C, cytomegalovirus, H. Pylori, and/or HIV in at-risk populations or when there is no other explanation)

Genetic screening for inherited thrombocytopenia and bone marrow failure syndromes and complex immunodeficiency diseases (4C). 

Recommendation exist against routine testing for H. pylori in children with persistent or chronic ITP unless they are in an at-risk population (1B).

  1.13. When to suspect cyclic thrombocytopenia?

Cyclic thrombocytopenia should be suspected in children diagnosed with chronic ITP not responsive to therapy with typical pattern of periodic platelets cycling on frequent platelet count monitoring (C).

2. Treatment of isolated thrombocytopenia
  2.1. What is the initial treatment of bleeding in a neonate with FNAIT?

Platelets should be transfused immediately if life-threatening bleeding is present (1B).

In the presence of life-threatening bleeding such as intracranial or gastrointestinal bleeding, platelets should be transfused to maintain platelet counts initially above 100 × 10^9^/L and then above 50 × 10^9^/L for at least 7 days (1D).

If an ICH is suspected clinically, do not delay platelet transfusion or initiating treatment while awaiting confirmation by imaging studies (1D).

In the absence of life-threatening bleeding in a neonate, such as intracranial or gastrointestinal bleeding, random donor platelets should be transfused to maintain a platelet count above 30 × 10^9^/L (1D).

Infuse the neonate with a count < 30 × 10^9^/L, with a random donor platelet transfusion and IVIG 1 g/kg (1C).

  2.2. What are the indications of platelet transfusion in a neonate with thrombocytopenia?

For preterm neonates with very severe thrombocytopenia (platelet count below 25 × 10^9^/L), platelet transfusions should be administered in addition to treating the underlying cause of the thrombocytopenia.

If the platelet count < (25 × 10^9^/L), transfuse in neonates with no bleeding.

If the platelet count < (50 × 10^9^/L), transfuse in neonates with bleeding, current coagulopathy, before surgery, or infants with FNAIT if previously affected sibling with ICH.

If the platelet count < (100 × 10^9^/L), transfuse in neonates with major bleeding or requiring major surgery (e.g., neurosurgery) (2C).

  2.3. What is the management of a neonate of mother with ITP?
    (1) Management of delivery

Cordocentesis and fetal scalp blood sampling should be avoided (C).

The mode of delivery should be determined by obstetric indications, not by anticipation of the neonatal platelet count (B).

Procedures during labor that may be associated with increased hemorrhagic risk to the fetus should be avoided, specifically the use of fetal scalp electrodes, fetal blood sampling, ventose delivery, and rotational forceps (C).

    (2) Management after delivery

The umbilical cord platelet count should be obtained at the time of delivery or as soon as possible (C).

FNAIT should be excluded by parental testing if the neonate presents with severe thrombocytopenia (platelet count < 50,000/μL) (C).

Repeat the platelet count as needed depending on platelet levels, trends in the count, and response to treatment (if any). If the cord platelet count is <100 × 10^9^/L, repeat the platelet count daily until stable as it may fall after birth (C). 

If the platelet count is <50 × 10^9^/L at birth, perform a cranial ultrasound (C) and consider an MRI.

In the case of ICH, give IVIg, limited steroids, and prn platelet transfusion to a maintain platelet count > 100 × 10^9^/L for 1 week if possible and >50 × 10^9^/L for another week and initial platelet transfusion (C).

If there is symptomatic bleeding or if the platelet count is <30 × 10^9^/L, with or without platelet transfusion, give IVIg (C). 

If severe thrombocytopenia continues for >1 week in a breast-fed infant, consider pausing breastfeeding for a few days to see whether the platelet count increases (mother with ITP or FNAIT) (C).

  2.4. What are the indications of hospitalization in pediatric patients with ITP?

Any severe (grade 4) bleeding requires immediate hospital admission and treatment to increase platelet levels until bleeding has decreased (C).

Any moderate (grade 3) bleeding requires hospital review (C) and requires hospital admission and therapy.

Worsening bleeding or significant comorbidities (C).

Risk of ICH (e.g., head trauma or unexplained headaches); patients at higher risk for ICH include those with a history of moderate or severe bleed in the preceding 28 days, recent administration (within 8 h) of NSAIDs, and another clinically significant coagulopathy (e.g., von Willebrand disease) and any recent history of head trauma (C).

Parents are anxious about bleeding and do not believe that they can control (young child) or restrict (older child) their child’s activity and the platelet count is <20,000 (C).

Parents cannot be relied upon to bring the child back readily if there is an emergency (e.g., they live too far away, they cannot afford to return, there are additional social concerns) (C).

Child has not spontaneously improved and must be overly restricted in activities due to parental over-restraint. This might necessitate treatment (C).

Patients not admitted to the hospital should receive education from the ER and expedited follow-up with a hematologist (within 24 to 72 h of the diagnosis or disease relapse) (C).

  2.5. What is the initial treatment of pediatric patients with ITP?

    I. The watch-and-wait policy based on clinical classification

At diagnosis, children and adolescents with ITP and mild or even moderate bleeding on a pediatric bleeding assessment tool (grade 1–3) may be managed expectantly with parental consent, supportive advice and a 24 h contact point, irrespective of the platelet count (B). Grade 3 patients may benefit from admission and treatment.

    II. Children with newly diagnosed ITP who have non-life-threatening mucosal bleeding and/or diminished HRQoL can start with any of the 1st line therapy:

Prednisone (2–4 mg/kg/day; maximum, 120 mg daily, for 5–7 days) (time to initial response 4–14 days) (C). For patients receiving corticosteroids, the treating physician should ensure the patient is adequately monitored for potential side effects regardless of the duration or type of corticosteroid selected (see implementation tool) and/or to make sure the course of steroids is short.

Intravenous immunoglobulin: For patients where corticosteroids are contraindicated or otherwise not preferred or there is urgency to increase the count, intravenous immunoglobulin (IVIG) can be used (C).

IVIG in a single dose of 0.8 to 1.0 g/kg (time to initial response 1–3 days) (A, Ib).

A second dose of IVIg may be administered if there is a suboptimal initial response and/or ongoing bleeding (C).

  2.6. What are the indications of platelet transfusion in pediatric patients with thrombocytopenia?

    I. In non-immune thrombocytopenia (2C).

Platelets < 10 × 10^9^/L transfuse irrespective of signs of hemorrhage.(other factors such as planning for bone marrow transplantation, risk of alloimmunization, proximity to health care need to be put in consideration in decision making)

Platelets < 20 × 10^9^/L transfuse in insertion of a non-tunneled central venous line.

Platelets < 50 × 10^9^/L transfuse in:

Moderate hemorrhage (e.g., gastrointestinal bleeding).

Surgery, unless minor (except at critical sites).

Platelets < 75–100 × 10^9^/L transfuse in:

Major hemorrhage or significant post-operative bleeding.

Surgery at critical sites: central nervous system including eyes.

In immune thrombocytopenia:

Patients with immune thrombocytopenia should only be transfused with platelets for life-threatening and possibly grade 3 bleeding (2B) or impending such bleeding, e.g., after major trauma or if on anticoagulation or antiplatelet agents or pre-op if the surgery is urgent or the patient’s platelets are unresponsive to medical therapy.

  2.7. What is the treatment of life-threatening bleeding in pediatric patient with thrombocytopenia?

For children with immune thrombocytopenia:

Combination therapy including IV corticosteroids, IVIg, with platelet transfusion.

Dose: IV methylprednisolone: 30 mg/kg per day, IVIg: 0.8–1.0 g/kg/d.

A second dose of IVIg and IV steroids is often required to sustain a platelet response or if one is not seen within 24 h of the initial dose (C).

IV anti-D 75 micrograms/kg (if available)can be given to Rh+, DAT- patients who are not too anemic.

A single dose of vincristine 0.03 mg/kg can be given.

Platelet transfusion (C) (bolus followed by continuous infusion if bleeding persists).

Antifibrinolytics may be given if bleeding continues despite therapy (C).

If there is an ICH, neurosurgical control of bleeding should be considered in conjunction with emergency platelet-raising therapy if there are signs of increased intracranial pressure (C).

Thrombopoietin receptor agonist (TPO-Ras) should be added to first-line treatments; they may aid the acute response in patients and especially prevent a decrease in the platelet count if initial response to emergency therapy decreases (C).

For children with non-immune thrombocytopenia:

Platelet transfusion is the main line of treatment (2B) (thrombopoietin receptor agonist (TPO-Ras) should be added to first-line treatments unless contraindicated).

What is the adjuvant treatment to stop bleeding in pediatric patients with thrombocytopenia?

Tranexamic acid (TXA) may be useful in certain dental or surgical procedures or a substantial risk for bleeding. Dose: 15–20 mg/kg every 8 h orally and e-aminocaproic acid 1–5 g every 4–6 h [maximum dose, 24 g/d] (D).

Hormonal therapy for heavy menstrual bleeding.

Antihistamine and moisturization for recurrent severe nosebleeds.

  2.8. What is the treatment of menorrhagia in adolescent girl with thrombocytopenia?

Manage as emergency treatment. Tranexamic acid can be useful and consult gynecologist for hormonal therapy (D). Prefer progesterone treatment over estrogen.

3. Prevention of bleeding in children and adolescents with isolated thrombocytopenia
  3.1. How to prevent serious bleeding in a fetus/neonate with FNAIT?
  At delivery:

- If the fetal platelet count is unknown, assisted delivery and invasive procedures on the fetus during delivery should be avoided, including forceps, vacuum-assisted delivery, scalp blood sampling and scalp electrodes (1D). Cesarian section is advised
- A cord blood sample should be sent for platelet count determination immediately after delivery (1C).
- Ideally, HPA-selected platelets should be available at the time of delivery (1C). Random platelets and IV Ig need to be available and are often effective.
  After delivery:
- In the absence of life-threatening bleeding in a neonate, such as intracranial or gastrointestinal bleeding, platelets should be transfused to maintain a platelet count above 30 × 10^9^/L (1D).

  3.2. How to prevent alloimmunization (maternal and transfusion related)?

In patients identified by screening or in sisters of patients with FNAIT, serial measurement of the concentration of HPA antibodies in subsequent pregnancies may be useful to determine the risk of FNAIT (2C).

Serial assessments of levels of anti-HPA-1a antibody in HPA-1a-not immunized women may be useful in identifying the risk of FNAIT(2C). Once they are sensitized as soon as the level increases, there is likely no further benefit to measuring titers.

Antenatal IVIG administration to the mother, commencing 1 g/kg/week at 12–16 weeks gestation, increase to 2 g/kg/week at 20 weeks or IVIG 1 g/kg/week at 12–16 weeks with the addition of corticosteroids at 1 mg/kg/day at 20 weeks or IVIG 0.5 g/kg/week at 12–16 weeks for the entire pregnancy or IVIG 2 g/kg/week at 12–16 or IVIG 2 g/kg/week at 12–16 weeks, add corticosteroids 1 mg/kg/day at 20 weeks should be suggested to all women in a subsequent pregnancy with maternal fetal incompatibility who have had a previous fetus or neonate with FNAIT-related ICH (1D).

If corticosteroids are used with IVIG, dexamethasone should not be used because of the associated risk of oligohydramnios (1C) and of effects on the fetus.

  3.3. What are the drugs to be avoided in a thrombocytopenic child with history of bleeding?

Salicylates,

NSAID, and

Anticoagulants (D).

Possibly also those associated with immune TP such as valproic acid, other antiepileptics.

  3.4. How to prevent bleeding in a thrombocytopenic child receiving antiplatelet medications?

Consider platelet transfusion to prevent bleeding in severe thrombocytopenia (platelet count < 10 × 10^9^/L) caused by abciximab or any other agent (abciximab is rarely used in children) (2C). When transfusing platelets to a child whose platelet function is not normal, twice as many platelets are needed as are needed in a thrombocytopenic patient.

  3.5. How to prevent further bleeding in a thrombocytopenic child following trauma?

Severely injured patients should be transported directly to an appropriate trauma facility (1B). 

The time between injury and bleeding control should be minimized (1A). 

Local compression is recommended to limit life-threatening bleeding (1A).

Use adjunct tourniquet to stop life-threatening bleeding from open extremity injuries in the pre-surgical setting (1B). 

Use adjunct pelvic binder to limit life-threatening bleeding in the presence of a suspected pelvic fracture in the pre-surgical setting (1B). 

Patients with an obvious bleeding source and those presenting with hemorrhagic shock in extremis and a suspected source of bleeding should undergo an immediate bleeding control procedure (1C). 

Use focused assessment with sonography in trauma (FAST) ultrasound for the detection of free fluid in patients with torso trauma (1C).

Early imaging using contrast-enhanced whole-body CT (WBCT) for the detection and identification of type of injury and potential source of bleeding is recommended (1B).

Laboratory screening of patients treated or suspected of being treated with anticoagulant agents should be performed (1C) and reversal agents considered.

Platelets should be administered to maintain a platelet count above 50 × 10^9^/L (1C).

Maintain a platelet count above 100 × 10^9^/L in patients with ongoing bleeding and/or traumatic brain injury (2C). 

Transfuse at an initial dose of four to eight single platelet units or one aphaeresis pack after Rh matching (2C).

Maintain a hemoglobin level of 70 to 90 g/L in patients with ongoing bleeding and/or traumatic brain injury (1C).

We recommend that TXA be administered to the trauma patient who is bleeding or at risk of significant hemorrhage as soon as possible and within 3 h after injury at a loading dose of 1 g infused over 10 min, followed by IV infusion of 1 g over 8 h (1A).

We recommend that protocols for the management of bleeding patients consider administration of the first dose of TXA on the way to the hospital (1C).

  3.6. How to assess risk of bleeding in children during preoperative evaluation?

Before surgery or invasive procedures, use a structured patient interview or standardized questionnaire which considers clinical and family bleeding and thrombotic history and detailed information on the patient’s medication.

Routine use of conventional coagulation screening tests such as activated partial thromboplastin time (aPTT), international normalized ratio (INR) and platelet count is not recommended in elective surgery, unless specific workup is indicated by the personal or family history or on examination.

We recommend the use of standardized questionnaires on bleeding and drug history as preferable to the routine use of conventional coagulation screening tests such as activated partial thromboplastin time (aPTT), international normalized ratio (INR) and platelet count in elective surgery (1 C).

In patients with normal platelet counts, preoperative platelet function testing is rarely useful and is suggested only in association with a positive bleeding history, decreased platelet function caused by medical conditions or antiplatelet medication (2B).

Bleeding time is not recommended for preoperative bleeding risk stabilization as it is influenced by many variables (C).

## 4. Discussion

This CPG was prepared to provide implementable consensus recommendations on the management of isolated thrombocytopenia in children and adolescents in Egypt. We followed an established methodology to systematically review the literature and applied the AGREE II tool to assess the adapted guidelines quality. Lastly, we mapped out CPG recommendations to identify underrepresented disease domains and topics of uncertainty to aid future research conduct.

There is a lack of evidence to support strong recommendations for various management approaches. Most of the adapted CPGs recommended complete clinical assessment, full blood count, and expert analysis of the peripheral blood smear at initial diagnosis to confirm a bleeding disorder, exclude secondary causes of thrombocytopenia and choose the type of work up required. According to the adapted guidelines, the International Society of Hemostasis and thrombosis–Bleeding assessment tool (ISTH-SCC BAT) could be used for initial screening of bleeding manifestations. Most of the included CPGs emphasized that the diagnosis of immune thrombocytopenic purpura (ITP) is based principally on exclusion of other causes of isolated thrombocytopenia. Given the lack of strong evidence for most of the recommendations in the pediatric age group, future research should focus on providing rigorous evidence on these management options in this vulnerable age group.

## 5. Strength and Limitations

To the best of our knowledge, this adapted guideline is the first to provide high-quality evidence about the management of idiopathic thrombocytopenia in children and adolescents in limited-resource countries like Egypt, providing applicable implementation tools.

However, this adapted guideline has some limitations. Since the AGREE II tool is a relatively recent tool, older CPGs may not have adopted the same standards, which may skew the findings, especially the section of stakeholder involvement in CPG development, including lay consumers, as well as the implementation in clinical practice section. This study was not able to assess the quality, strength and confidence of recommendations in the adapted guidelines homogenously as the evidence grading systems used in different CPGs was different. This undermined the ability to analyze the current evidence gap thoroughly. Moreover, the scarcity and poor quality of available primary studies on idiopathic thrombocytopenia in children and adolescents were reported as sources of heterogeneity and imprecision in the published CPGs. However, understanding this evidence gap could highlight the importance of providing more studies in this field of interest.

## 6. Conclusions

This adapted CPG provides consensus recommendations and implementation tools on the management of isolated thrombocytopenia in children and adolescents in Egypt. Current CPGs on the diagnosis and management of idiopathic thrombocytopenia in children and adolescents vary in their scope and methodological quality. Moreover, there is lack of strong evidence to support most of the adapted recommendations in the current CPGs and most of the evidence is taken from adult studies, which may hinder evidence translation into clinical practice. Thus, future research should aim at performing high-quality studies to provide sufficient evidence in the field of pediatric isolated thrombocytopenia. Moreover, a future study should report the outcome of this adapted guideline and include cost-analysis evaluations.

## Figures and Tables

**Table 1:** AGREE II Appraisals of the Source Clinical Practice Guidelines (CPGs).

	Domains							
	1	2	3	4	5	6	Overall	Is CPG Recommended for Use?
CPG1	95.9	100	100	95.9	89.7	92.6	95.7	Yes
CPG2	87.9	82.9	83	85.7	75	64.6	79.9	Yes
CPG3	77.4	77.4	73.7	100	73.7	73.7	79.3	Yes
CPG4	87.7	78.7	89.1	87.7	81.6	87.7	85.4	Yes
CPG5	89.7	84.4	89.1	89.7	79.6	89.7	87.0	Yes
CPG6	74.7	74.7	94.3	100	89.3	92.9	87.7	Yes
CPG7	95.6	96.9	97.5	100	83.2	89.7	93.8	Yes
CPG8	33.3	55.6	25	83.3	58.3	66.7	46	No
CPG9	33.3	77.8	22.9	83.3	66.7	66.7	50.1	No
CPG10	11.1	55.6	25	83.3	66.7	66.7	49.2	No
CPG11	33.3	77.8	22.9	55.6	58.3	66.7	45.7	No
CPG12	11.1	11.1	4.2	38.9	33.3	66.7	23.6	No

CPG1: American Society of Hematology 2019 guidelines for immune thrombocytopenia (ASH, 2019) [1]. CPG2: The European guideline on management of major bleeding and coagulopathy following trauma: fifth edition (European, 2019) [2]. CPG3: Management of severe perioperative bleeding: guidelines from the European Society of Anaesthesiology (ESA, 2016) [3]. CPG4: Fetal and neonatal alloimmune thrombocytopenia: recommendations for evidence-based practice, an international approach (BSH, 2019) [4]. CPG5: Guidelines on transfusion for fetuses, neonates and older children (BSH, 2016) [5]. CPG6: Guidelines for the Laboratory Investigations of heritable disorders of platelet function (BSH, 2011) [6]. CPG7: Updated international consensus report on the investigation and management of primary immune thrombocytopenia (ASH ITP consensus, 2019) [7]. CPG8: Guidelines on the investigation and management of thrombocytopenia in pregnancy and neonatal alloimmune thrombocytopenia (1996) [50]. CPG9: Platelet disorders in children: A diagnostic approach (2011) [51]. CPG10: A review of inherited platelet disorders with guidelines for their management on behalf of the UKHCDO (2006) [52]. CPG11: Fundamentals for a Systematic Approach to Mild and Moderate Inherited Bleeding Disorders: An EHA Consensus Report (2019) [55]. CPG12: Trust Guideline for the Management of Newly Diagnosed Immune Thrombocytopenia (ITP) in Children (2021) [56].

**Table 2:** PIPOH Model for Health/Clinical Questions.

**P (patients, target population):**
- ***Gender*:** Both genders.
- ***Age group*:** Neonates, infants, children, and adolescents less than 18 years.
- ***Disease/Condition*:** Bleeding (with focus on thrombocytopenia).
- ***Exclusion criteria*:** Platelet function defects, bone marrow failure, malignancy and chemotherapy-related thrombocytopenia, collagen disease and vascular disorders.
**I (interventions and practices considered/guideline category):**
- ***Diagnosis:***
▪ Clinical: History and examination
▪ Laboratory diagnosis
- ***Treatment:***
▪ During bleeding episode
▪ Between the bleeding episodes
- ***Prevention:***
▪ Primary prevention
▪ Secondary prevention
**P (Professionals/intended or target users and clinical specialties):**
- Primary health care physicians at Ministry of Health (MOH)
- General practitioners
- Family medicine specialists
- Pediatricians
- Hematologists
- Nurses
- Medical students
- Laboratory hematologists and blood bank specialists
- Neonatologists
- Obstetricians and gynecologists
**O (major outcomes considered):**
- Primary outcome
- Prevent mortality
- Prevent morbidity from thrombocytopenia, e.g., bleeding, impaired HRQoL
- Prevent sequelae and disabilities
- Secondary outcome
- Family counseling
- Prevention of further episodes
- H (Health care settings)
- Type: Primary and secondary health care setting: outpatient clinic, emergency room
- Health care sector: Governmental, non-governmental and private sectors

**Table 3:** Level of Evidence.

Evidence Level	Definition
Ia	Evidence obtained from meta-analysis of RCTs
Ib	Evidence obtained from ≥1 RCT
IIa	Evidence obtained from ≥1 well-designed controlled study without randomization
IIb	Evidence obtained from ≥1 other type of well-designed quasi-experimental study
III	Evidence obtained from well-designed non-experimental descriptive studies, such as comparative studies, correlated studies, and case studies
IV	Evidence obtained from expert committee reports or opinions and/or clinical experience of respected authorities

RCT, randomized controlled trial. Reprinted with permission from [7].

**Table 4:** Grading of recommendation.

Grade of Recommendation	Definition	Level of Evidence
A	Requires ≥ 1 RCT as part of a body of literature of overall good quality and consistency addressing specific recommendation	Evidence levels Ia and Ib
B	Requires the availability of well-conducted clinical studies but no randomized clinical trials on the topic of recommendation	Evidence levels IIa, IIb and III
C	Requires evidence obtained from expert committee reports or opinions and/or clinical experiences of respected authorities. Indicates an absence of directly applicable clinical studies of good quality	Evidence level IV

RCT, randomized controlled trial. Reprinted with permission from [7].

## Data Availability

The data presented in this study are available on request from the corresponding author due to privacy.

## Supplementary Materials

The following supporting information can be downloaded at: https://www.mdpi.com/article/10.3390/children11040452/s1, Table S1. The RIGHT-Ad@pt checklist.

## Abbreviations

aPTT	Activated partial thromboplastin time
AGREE II	Appraisal of Guidelines for REsearch and Evaluation (Version II)
CPG	Clinical practice guideline
CBC	Complete blood count
DAT	Direct antibody test
EPG	Egyptian Pediatric Clinical Practice Guidelines Committee
FNAIT	Fetal/neonatal alloimmune thrombocytopenia
FAST	Focused assessment with sonography in trauma
HRQoL	Health related quality of life
ITP	Immune thrombocytopenic purpura
Ig	Immunoglobulin
INR	International normalized ratio
IVIg	Intravenous immunoglobulins
ICH	Intracranial hemorrhage
ISTH-SCC BAT	International Society of Hemostasis and thrombosis–Bleeding assessment tool
NAIT	Neonatal allo-immune thrombocytopenia
NSAIDs	Non-steroidal anti-inflammatory drugs
PREPARE	Practice Guideline REgistry for transPAREncy
TXA	Tranexamic acid
TPO-Ras	Thrombopoietin receptor agonist
WBCT	Whole-body CT
WHO	World Health Organization
