# Supplementary material for: Egyptian Pediatric Guidelines for the Management of Children with Isolated Thrombocytopenia Using the Adapted ADAPTE Methodology—A Limited-Resource Country Perspective
Source: Children (Basel). 2024 Apr 9;11(4):452. doi: 10.3390/children11040452 (PMC11048986; doi:10.3390/children11040452)
Supplement: Supplementary file 1 [file children-11-00452-s001.zip › children-2890055-supplementary.pdf]

Table S1. The RIGHT-Ad@pt checklist

7 sections, 27 topics, and 34 items		Assessment	Page(s)*	Note(s)
**BASIC INFORMATION**				
**Title/subtitle**				
1	Identify the report as an adaptation of practice guideline(s), that is include "guideline adaptation", "adapting", "adapted guideline/recommendation(s)", or similar terminology in the title/subtitle.	☒ Yes<br>☐ No<br>☐ Unclear		
2	Describe the topic/focus/scope of the adapted guideline.	☒ Yes<br>☐ No<br>☐ Unclear		
**Cover/first page**				
3	Report the respective dates of publication and the literature search of the adapted guideline.	☒ Yes<br>☐ No<br>☐ Unclear		
4	Describe the developer and country/region of the adapted guideline.	☒ Yes<br>☐ No<br>☐ Unclear		
**Executive summary/abstract**				
5	Provide a summary of the recommendations contained in the adapted guideline.	☒ Yes<br>☐ No<br>☐ Unclear		
**Abbreviations and acronyms**				
6	Define key terms and provide a list of abbreviations and acronyms (if applicable).	☒ Yes<br>☐ No<br>☐ Unclear		
**Contact information of the guideline adaptation group**				
7	Report the contact information of the developer of the adapted guideline.	☒ Yes<br>☐ No<br>☐ Unclear		
**SCOPE**				
**Source guideline(s)**				
8	Report the name and year of publication of the source guideline(s), provide the citation(s), and whether source authors were contacted.	☒ Yes<br>☐ No<br>☐ Unclear		
**Brief description of the health problem(s)**				
9	Provide the basic epidemiological information about the problem (including the associated burden), health systems relevant issues, and note any relevant differences compared to the source guideline(s).	☒ Yes<br>☐ No<br>☐ Unclear		
**Aim(s) and specific objectives**				
10	Describe the aim(s) of the adapted guideline and specific objectives, and note any relevant differences compared to the source guideline(s).	☒ Yes<br>☐ No<br>☐ Unclear		
**Target population(s)**				
11	Describe the target population(s) and subgroup(s) (if applicable) to which the recommendation(s) is addressed in the adapted guideline, and note any relevant differences compared to the source guideline(s).	☒ Yes<br>☐ No<br>☐ Unclear		
**End-users and settings**				
12	Describe the intended target users of the adapted guideline, and note any relevant differences compared to the source guideline(s).	☒ Yes<br>☐ No<br>☐ Unclear		
13	Describe the setting(s) for which the adapted guideline is intended, and note any relevant differences compared to the source guideline(s).	☒ Yes<br>☐ No<br>☐ Unclear		

7 sections, 27 topics, and 34 items	Assessment	Page(s)*	Note(s)
**RIGOR OF DEVELOPMENT**			
**Guideline adaptation group**			
14 List all contributors to the guideline adaptation process and describe their selection process and responsibilities.	☒ Yes<br>☐ No<br>☐ Unclear		
**Adaptation framework/methodology**			
15 Report which framework or methodology was used in the guideline adaptation process.	☒ Yes<br>☐ No<br>☐ Unclear		
**Source guideline(s)**			
16 Describe how the specific source guideline(s) was(were) selected.	☒ Yes<br>☐ No<br>☐ Unclear		
**Key questions**			
17 State the key questions of the adapted guideline using a structured format, such as PICO (population, intervention, comparator, and outcome), or another format as appropriate.	☒ Yes<br>☐ No<br>☐ Unclear		
18 Describe how the key questions were developed/modified, and/or prioritized.	☐ Yes<br>☒ No<br>☐ Unclear		
**Source recommendation(s)**			
19 Describe how the recommendation(s) from the source guideline(s) was(were) assessed with respect to the evidence considered for the different criteria, the judgements and considerations made by the original panel.	☐ Yes<br>☒ No<br>☐ Unclear		
**Evidence synthesis**			
20 Indicate whether the adapted recommendation(s) is/are based on existing evidence from the source guideline(s), and/or additional evidence.	☒ Yes<br>☐ No<br>☐ Unclear		
21 If new research evidence was used, describe how it was identified and assessed.	☐ Yes<br>☒ No<br>☐ Unclear	NA	
**Assessment of the certainty of the body of evidence and strength of recommendation**			
22 Describe the approach used to assess the certainty/quality of the body/ies of evidence and the strength of recommendations in the adapted guideline and note any differences (if applicable) compared to the source guideline(s).	☐ Yes<br>☒ No<br>☐ Unclear	NA	
**Decision-making processes**			
23 Describe the processes used by the guideline adaptation group to make decisions, particularly the formulation of recommendations.	☒ Yes<br>☐ No<br>☐ Unclear		
**RECOMMENDATIONS**			
**Recommendations**			
24 Report recommendations and indicate whether they were adapted, adopted, or *de novo*.	☒ Yes<br>☐ No<br>☐ Unclear		
25 Indicate the direction and strength of the recommendations and the certainty/quality of the supporting evidence and note any differences compared to the source recommendations(s) (if applicable).	☒ Yes<br>☐ No<br>☐ Unclear		
26 Present separate recommendations for important subgroups if the evidence suggests important differences in factors influencing recommendations and	☒ Yes<br>☐ No<br>☐ Unclear		

7 sections, 27 topics, and 34 items	Assessment	Page(s)*	Note(s)
note any differences compared to the source recommendations(s) (If applicable).			
**Rationale/explanation for recommendations**<br>27 Describe the criteria/factors that were considered to formulate the recommendations or note any relevant differences compared to the source guideline(s) (if applicable).	☐ Yes<br>☒ No<br>☐ Unclear		
**EXTERNAL REVIEW AND QUALITY ASSURANCE**			
**External review**<br>28 Indicate whether the adapted guideline underwent an independent external review. If yes, describe the process.	☒ Yes<br>☐ No<br>☐ Unclear		
**Organizational approval**<br>29 Indicate whether the adapted guideline obtained organizational approval. If yes, describe the process.	☒ Yes<br>☐ No<br>☐ Unclear		
**FUNDING, DECLARATION, AND MANAGEMENT OF INTEREST**			
**Funding source(s) and funder role(s)**<br>30 Report all sources of funding for the adapted guideline and source guideline(s), and the role of the funders.	☒ Yes<br>☐ No<br>☐ Unclear		
**Declaration and management of interests**<br>31 Report all conflicts of interest of the adapted and the source guideline(s) panels, and how they were evaluated and managed.	☒ Yes<br>☐ No<br>☐ Unclear		
**OTHER INFORMATION**			
**Implementation**<br>32 Describe the potential barriers and strategies for implementing the recommendations (if applicable).	☒ Yes<br>☐ No<br>☐ Unclear		
**Update**<br>33 Briefly describe the strategy for updating the adapted guideline (if applicable).	☒ Yes<br>☐ No<br>☐ Unclear		
**Limitations and suggestions for further research**<br>34 Describe the challenges of the adaptation process, the limitations of the evidence, and provide suggestions for future research.	☐ Yes<br>☒ No<br>☐ Unclear	NA	
